# A Single-Event-Hardened Scheme of Phase-Locked Loop Microsystems for Aerospace Applications

**DOI:** 10.3390/mi13122102

**Published:** 2022-11-28

**Authors:** Qi Xiang, Hongxia Liu, Yulun Zhou

**Affiliations:** Key Laboratory for Wide Band Gap Semiconductor Materials and Devices of Education Ministry, School of Microelectronics, Xidian University, Xi’an 710071, China

**Keywords:** aerospace, phase-locked loop, single event transient, radiation-hardened-by-design

## Abstract

In order to improve the ability of the phase-locked loop (PLL) microsystem applied in the aerospace environment to suppress the irradiation effect, this study presents an efficient charge pump hardened scheme by using the radiation-hardened-by-design (RHBD) technology. In this study, the sensitivity analysis of the single-event transient (SET) at different nodes of charge pump and different bombardment energies is carried out. Without changing the original structure and loop parameters, a hardened scheme of phase-locked loop to suppress the single-event effect is proposed. A digital control circuit is added between the charge pump and low-pass filter, which greatly reduces the sensitivity of the charge pump to the SET. The classical double-exponential current pulse model is used to simulate the SET effect on the unreinforced and reinforced phase-locked loops, and the reliability of the proposed reinforcement scheme is verified. The simulation results based on the SMIC 130 nm standard complementary metal–oxide–semiconductor (CMOS) process show that the peak value of the transient response fluctuation of the phase-locked loop using the proposed single-event-hardened scheme decreased by 94.2%, the lock recovery time increased by 75.3%, and the maximum phase shift decreased by 90.8%. This shows that the hardened scheme can effectively reduce the sensitivity of the PLL microsystems to the SET effects.

## 1. Introduction

**Figure 1:**
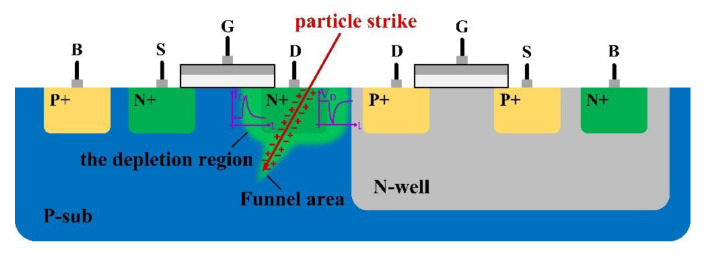
The Influence Mechanism of SET on CMOS Devices.

As a classical mixed signal circuit, the PLL is widely used in clock generator and other circuits [1], due to its simple structure and high stability, and is the core component of a spacecraft’s electronic system [2]. A single-event transient is the most common single-event effect, and its impact on devices is shown in Figure 1. When high-energy particles bombard semiconductor devices, the charge generated by the ionization will be collected by the device source and drain, forming a transient current pulse at the electrode, thus affecting the normal operation of the circuit [3]. The PLL working in the irradiation environment is prone to be bombarded by high-energy particles, which induces single-event transient, causing phase and frequency drift of the output signal, leading to chaos in the entire communication system of the spacecraft and seriously threatening the reliability of the spacecraft [4,5,6]. Each module constituting the PLL has different responses to the SET effect [7,8,9]. Previous research results have shown that the charge pump (CP) is the most sensitive part of the phase-locked loop. When the charge pump circuit is exposed to the radiation environment, the error rate generated is at least two orders of magnitude higher than in other modules [10,11,12]. Therefore, in order to improve the ability of the PLL system to suppress the SET effect, it is necessary to carry out an effective hardened design for the CP.

In recent years, in order to reduce the effect of the SET on a PLL, researchers have proposed various methods to reduce the SET sensitivity of the CP output stage [2,13,14,15,16,17]. Loveless et al. [15] proposed that the hardened scheme of changing the current-type CP to the voltage-type CP could effectively reduce the SET sensitivity. The main principle was to reduce the sensitive nodes in the current-type CP, improve the charge–discharge rate, and accelerate the PLL locking time; however, this changes the loop parameters of the system and increases the PLL jitter. Zhao et al. [16] used a complementary current-limiting circuit consisting of a pair of complementary operational amplifiers, a resistor, and a pair of complementary SET-current-limiting transistors. The output current of the charge pump was constantly detected through a resistor. When the current exceeded the set threshold, the operational amplifier was started to turn on the current-limiting transistor to generate a compensation current, limiting the current change until the current dropped below the threshold. This scheme effectively suppressed the SET effect of the CP; however, the introduction of a large resistance affected the dynamic characteristics of the PLL system and reduced the phase noise. Han et al. [17] proposed a charge pump compensation structure consisting of a latch detection circuit, two operational amplifiers, and four MOS devices. They used the resistance in the low-pass filter (LPF) to detect the transient current and then used a charge-compensation circuit to compensate the charge to the LPF until the voltage on the LPF returned to the original value. However, this scheme only shortened the recovery time; it did not reduce the maximum phase shift, and it increased the area of the compensation circuit. At present, many CP hardened schemes have introduced amplifiers or comparators for SET current detection. When the introduced structure itself is impacted by high-energy particles, it brings additional charges to the phase-locked loop system and improves the system’s SET sensitivity. In addition, the hardened scheme should also be considered in the compromise of area and power consumption, rather than changing the circuit parameters.

In this work, in order to reduce the phase-locked loop’s sensitivity to SET, a single-event-hardened scheme for the charge pump is proposed, and a standard 130 mm CMOS process is used to test the performance of the phase-locked loop and verify the reliability of the hardened scheme. The next section describes the PLL architecture, the current pulse establishment of the SET effect, and the analysis of the charge pump’s SET sensitivity; the third section describes the working mechanism of the SET hardened structure of the charge pump in detail. The fourth section is the simulation of the SET effect on unhardened and hardened PLLs and the analysis of the simulation results. The last section is the summary of the work.

## 2. Responses of SET in CP

### 2.1. Topology of the CPPLL

**Figure 2:**
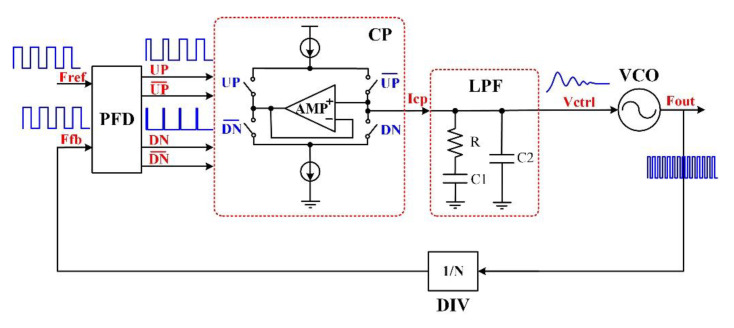
The topology of the CPPLL.

The phase-locked loop is a feedback control system that compares the feedback signal with the input reference signal. It is often used to achieve frequency synthesis, clock generation, clock recovery, and other functions [18]. Figure 2 shows the topological structure of the charge pump phase-locked loop (CPPLL), which is mainly composed of five submodules, namely, the frequency discriminator (PFD), the charge pump (CP), the low-pass filter (LPF), the voltage-controlled oscillator (VCO), and the frequency divider (DIV). The PFD detects the phase or frequency of the input reference signal Fref and the feedback signal Ffb after frequency division and generates the CP charge–discharge control signals UP and DN according to the phase difference or frequency difference. The charge pump charges or discharges the LPF under the control of the UP and DN, changing the output voltage of the low-pass filter. By adjusting the control voltage Vctrl of the VCO, the output frequency of the VCO is changed, and the phase difference between the input reference signal Fref and the feedback signal Ffb is reduced. This feedback process repeats continuously until the phase of the input reference signal and the feedback signal is finally aligned, realizing CPPLL locking and outputting a continuously stable N * Ffb frequency signal.

**Figure 3:**
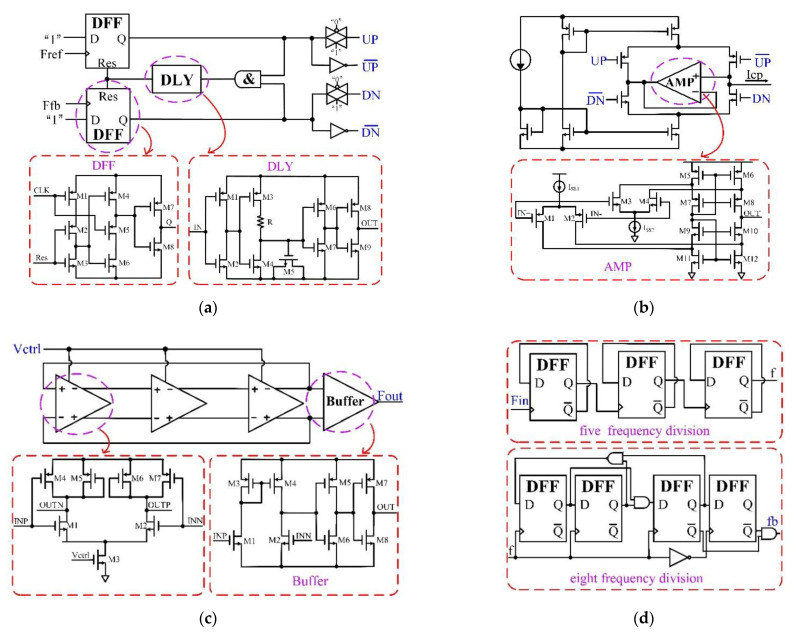
This work uses the submodule circuit of a CPPLL: (**a**) topology of three-state frequency and phase detector based on D-flip-flop; (**b**) topology of charge pump with unity gain amplifier; (**c**) topology of voltage-controlled oscillator cascaded with three differential-delay units; (**d**) topology of frequency divider cascaded with D-flip-flop.

The submodules used by the phase-locked loop designed in this work are shown in Figure 3. The PFD module adopts the classical three-state frequency and phase difference circuit structure based on D flip-flop, compares the phase difference and frequency difference of the reference signal and the feedback signal, converts them into error-related square wave signals, and inputs them into the CP circuit. In order to eliminate the influence of non-ideal factors such as charge sharing, the CP module uses a charge pump structure with a unity gain amplifier. The CP converts the PFD output signal into a charging and discharging current, charges and discharges the capacitor in the loop filter, and filters it; it becomes the input to the VCO module. The VCO module adopts a ring oscillator structure. Considering the tradeoff among the response speed, power consumption, and noise parameters of the oscillator, this paper adopts a voltage-controlled oscillator circuit, which cascades three differential-delay units to realize the conversion between the output voltage and the output frequency of the filter. DIV uses the form of five-frequency division and eight-frequency division cascaded; that is, the division ratio N is 40. The frequency of the VCO output signal is divided and fed back to the PFD for comparison with the output reference frequency. The whole PLL system circuit is connected as an integration loop.

### 2.2. Analysis of SET’s Impact on CP

The number of error clock pulses generated by a CP bombarded by high-energy particles is at least two orders of magnitude more than that of other modules bombarded. Therefore, the CP is recognized as the module that is most sensitive to the SET effect in a PLL. This section adopts the method of double-exponential pulse current injection to the charge pump circuit node to simulate the SET bombardment and simulates and analyzes the SET sensitivity of the charge pump under different bombardment nodes and different bombardment energies.

#### 2.2.1. SET Current Pulse Model

In the circuit level simulation of a single-event effect, the influence of the SET effect on the circuit can be simulated by an instantaneous current pulse. In this work, the classic double-exponential current pulse model is used to simulate the influence of a SET effect. The double-exponential current pulse model was proposed by Messenger et al. [19] after simplified analysis based on the physical mechanism of the single-event effect. The analytical equation of the double-exponential current source is

(1) $$I\left(t\right)=\frac{Q_{tot}}{\tau_{a}-\tau_{b}}\left(e^{\frac{-t}{\tau_{a}}-\frac{-t}{\tau_{b}}}\right)$$

In Equation (1), $Q_{tot}$ is the total charge generated by ionization when high-energy particles enter the device, $\tau_{a}$ is the charge collection constant, and $\tau_{b}$ is the time constant of the ionized electron-hole trajectory. The physical models of the MOS devices in SMIC130 nm standard CMOS process were built in TCAD software. The single-event effect simulation was conducted on the physical model, and the obtained transient current was fitted to calibrate the time constant. By adjusting the value of $Q_{tot}$, the transient current generated by the incident high-energy particles with different energies was obtained. In this work, a 500 fC deposition charge was used to verify the single-event transient effect. The peak current of the fault pulse was 2.1 mA, the rise time $\tau_{a}$ was 12 ps, and the fall time $\tau_{b}$ was 216 ps.

#### 2.2.2. SET Response of Different Bombardment Nodes

**Figure 4:**
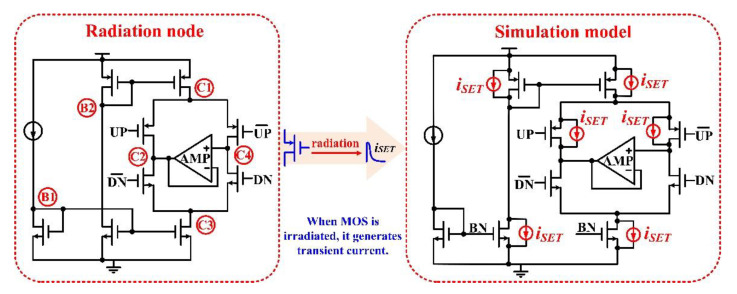
SET circuit level simulation for different nodes of the CP.

When high-energy particles enter the sensitive node of the circuit, the electron–hole pairs generated by ionization will be collected by the circuit node and generate a large transient current. In the circuit level simulation of the single-event effect, the current model was injected into the sensitive node to simulate the bombardment of high-energy particles on the circuit. As shown in Figure 4, double-exponential current pulse bombardment experiments were conducted on all nodes of the classical charge pump structure, where B1 and B2 are nodes in the charge pump bias circuit, and C1~C4 are nodes of the charge pump OP-Amp.

**Figure 5:**
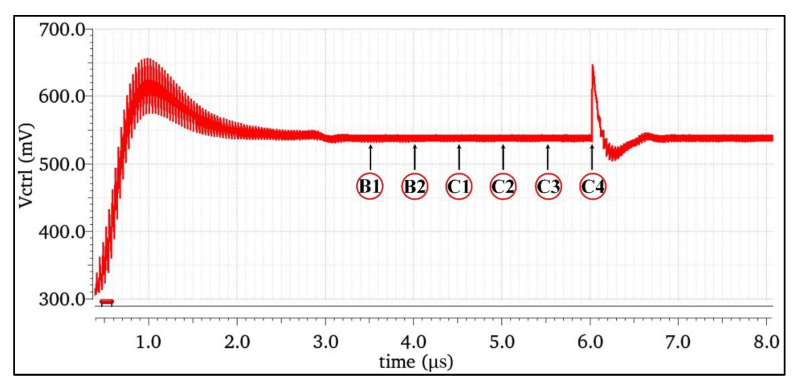
Simulation results of the SET responses of different nodes in a charge pump.

Figure 5 shows the output results of each node of the charge pump after being bombarded by a SET. It can be seen that when a node in the bias circuit was bombarded by a single-particle transient current, the control voltage produced less disturbance and was less sensitive to the SET. The main reason was that the high-frequency voltage disturbance of the bias circuit was filtered by the node capacitance on the transmission path, when it was transmitted to the LPF through the CP, resulting in a large degree of attenuation. When the OP-Amp node was bombarded, there was obvious disturbance, and it was more sensitive to the SET. The output node C4 was the most sensitive node. The main reason was that the transient current pulse generated by the output stage node directly charged or discharged the LPF capacitor, resulting in a large transient of the LPF output voltage.

#### 2.2.3. SET Response of Different Bombardment Energy

**Figure 6:**
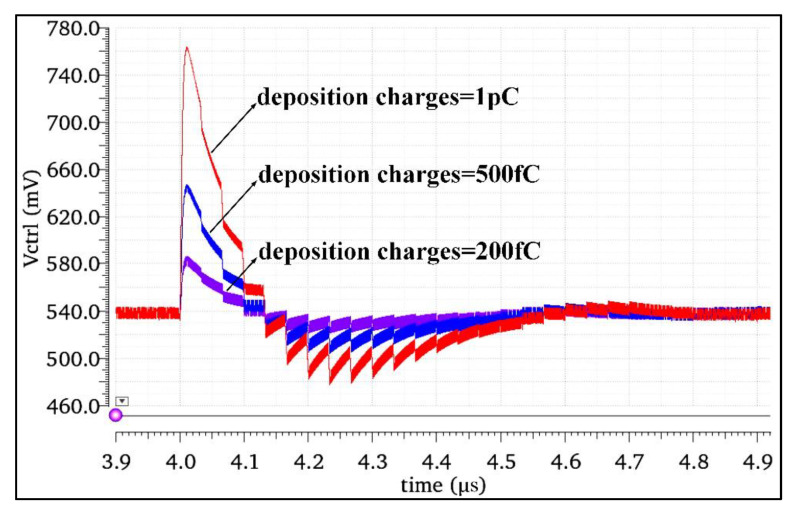
Simulation results of the response to the SET with different bombardment energies in the charge pump.

The above section determined that the output node C4 was the most sensitive node in the CP circuit through simulation. This section selected node C4 to study the impact of different bombardment energies on the CP. We injected the double-exponential current pulses corresponding to the high-energy particles with deposition charges of 200 fC, 500 fC, and 1 pC into node C4, respectively, to obtain the impact of different bombardment energies on the Vctrl, as shown in Figure 6.

The simulation results in Figure 6 show that the greater the energy of the incident high-energy particles, the more obvious the SET effect of the charge pump was: the increase in the incident energy led to an increase in the amount of charges deposited in the circuit nodes, causing greater disturbance to the node voltage, and the circuit needed a longer time to release or replenish the charges, resulting in a greater phase shift of the output signal of the frequency synthesizer.

## 3. The Proposed SET Hardened CP Circuit

The CP is a switch controlled by the PFD output signal. When the complementary switch is bombarded by high-energy particles, the switch opens abnormally, and charge will flow in or out. At the same time, the CP is directly connected to the LPF. When the single-event effect directly acts on the connected output MOS tube, the generated deposition charge is directly transferred to the LPF. Based on the above effects of the high-energy particles on the CP circuit, this work added a digital control circuit between the CP and the LPF to cut off the SET current transmitted to the LPF module, release the charges generated by the SET effect through the charge release path, real-time suppress the effects of single-particle radiation, and improve the irradiation resistance of the CP circuit.

**Figure 7:**
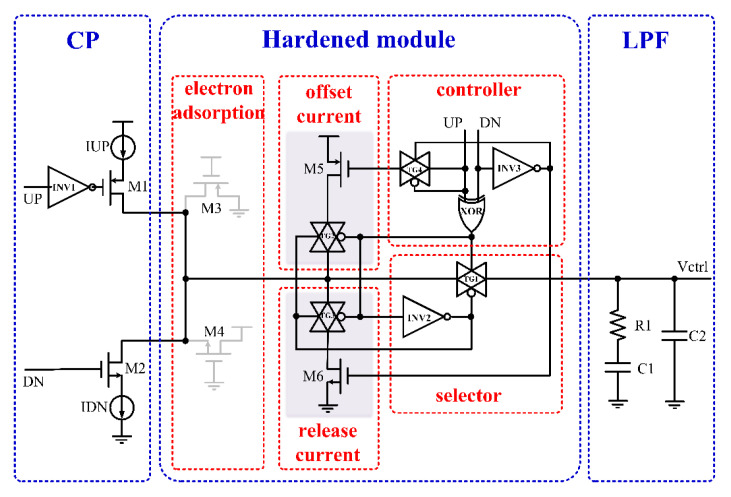
The proposed SET-hardened CP.

The circuit structure of the single-event-hardened charge pump proposed in this work is shown in Figure 7. A digital control circuit composed of an exclusive-OR gate (XOR gate), transmission gate, inverter, and MOS switch was added between the CP and LPF circuit to suppress the influence of the SET effect. This digital control circuit distinguished between the unlocked state and locked state of the PLL. When the PLL was in the unlocked state, the digital control circuit turned on the TG1, turned off the TG2 and TG3, and the PLL worked normally. When the PLL was locked, the digital control circuit turned off the TG1, blocked the path from the CP to the LPF and the VCO, and reduced the influence of the transient current generated by the high-energy particles bombarding the CP on the LPF and VCO, thus reducing the change value of the control voltage of the voltage-controlled oscillator. At the same time, the digital control circuit turned on the TG2 and TG3 and added an additional current source to assist the charge pump current to discharge the deposited charge, thus reducing the recovery time of the PLL’s return to normal frequency. The detailed reinforcement principle of the proposed reinforcement circuit is as follows: when UP = 0, DN = 1 or UP = 1, DN = 0, the PLL is in an unlocked state. The high level generated by the XOR in the control module causes the TG1 of the selection module to turn on, and the TG2 of the compensation current model and the TG3 of the release current module are turned off. The charging and discharging current can normally charge or discharge the LPF through TG1. At this time, if the CP is affected by the SET effect, but the PLL is unlocked, the effect is eliminated through the feedback mechanism. When UP = 0 and DN = 0, the PLL is locked, the TG1 of the selection module is off, and the TG2 of the compensation current model and the TG3 of the release current module are turned on. When the TG1 disconnects the CP from the LPF, the charge generated by the SET effect of the CP is released by the M5 or M6 tube after the TG2 or TG3 is turned on, which does not affect the LPF module. It should be noted that when the PLL is in lock, the TG1 is off and the CP will disconnect with the LPF and VCO, which means that the VCO has no feedback mechanism, so the output phase and frequency can be easily changed. However, when there is a phase difference between the output phase and the input reference signal after the output phase changes, the PFD outputs the control signals, UP and DN pulses, when it detects the phase difference, so that the control signals are UP = 0, DN = 1 or UP = 1, DN = 0, and the transmission gate TG1 is turned on. Under the adjustment of the feedback mechanism, the frequency and phase of the output signal can be guaranteed to be stable, but this increases the fluctuation value of the output frequency of the PLL. Therefore, it is necessary to reduce the locking time of the PLL and increase the bandwidth of the PLL, so that the PLL can respond faster and adjust the control voltage quickly through the feedback mechanism to ensure the stability of the output phase and frequency. In addition, the M3 and M4 of the charge absorption module have the function of absorbing the SET charges and can effectively absorb the charges generated by the SET hitting the drain of the switch tube. At the same time, the two MOS tubes are not on and do not affect the performance of the circuit.

In this work, additional digital control circuits were added to improve the radiation resistance of the circuit, so the added circuit itself should have a strong radiation resistance. The differential cascade voltage switch logic (DCVSL) is equivalent to a latch unit, and the output is controlled by two node voltages [20]. It has strong resistance to a SET. The DCVSL structure was adopted for the XOR gate, transmission gate, and inverter in the charge pump circuit, which gave the gate circuit strong resistance to the SET.

## 4. Simulation and Result Analysis

### 4.1. Verification of Basic Performance

**Figure 8:**
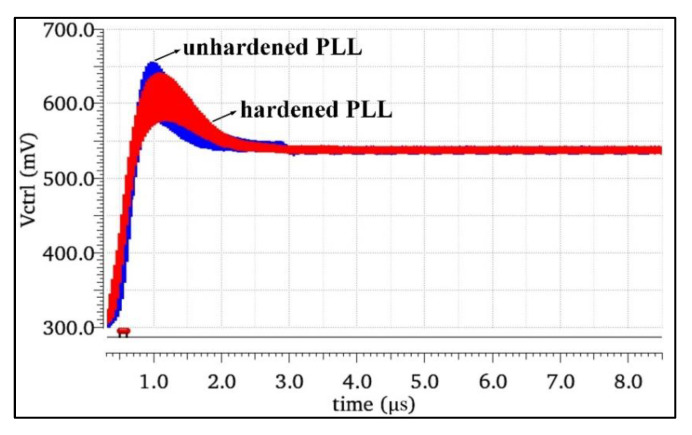
Locking processes of the RHPLL and NHPLL.

The SMIC130nm CMOS standard process was used to build the unhardened PLL (NHPLL) and hardened PLL (RHPLL) systems, respectively. Except for the CP, the rest of the two circuits were identical. The basic characteristics of the two PLLs before and after reinforcement were simulated to verify that the reinforcement structure had no significant impact on the basic performance of the system. Through the overall simulation of the CPPLL before and after the reinforcement, the locking state was obtained as shown in Figure 8. The simulation results showed that the locking times of the NHPLL and RHPLL were 2.3 μs and 2.5 μs, respectively, and the PLL system with the charge pump hardened did not change its loop performance.

**Figure 9:**
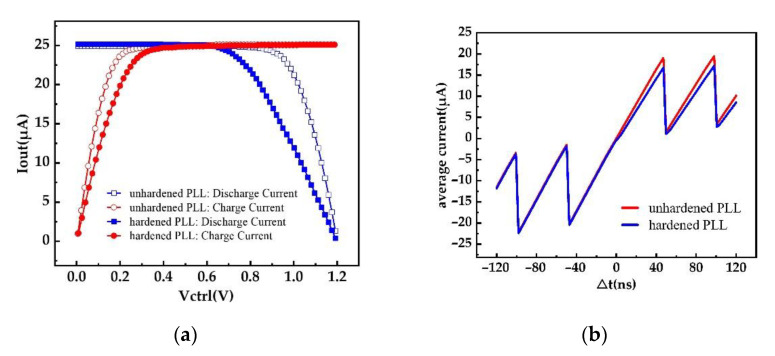
(**a**) Current mismatch of the RHPLL and NHPLL. (**b**) Current linearity of the RHPLL and NHPLL.

The PLL hardened scheme proposed in this work was for the charge pump part; so, it was necessary to simulate the overall charge–discharge current matching of the CP and verify the influence of the reinforcement structure on the current matching. The simulation results of the charge–discharge current mismatch of the two PLLs are shown in Figure 9a. The results showed that the current matching degree of the hardened PLL decreased slightly. Figure 9b shows the simulation result of the current linearity, and the linearity of the RHPLL also decreased slightly. The decrease in the matching and linearity was caused by the transmission gate TG1 in the hardened structure. The parasitic capacitance of the TG1 reduced the linearity of the charge pump charge–discharge current, leading to the decrease in the CP charge–discharge current matching. However, the simulation results also showed that when the control voltage was 550 mV, the two PLLs had good matching. Therefore, when the control voltage was controlled at 550 mV, the slight decrease in the current matching caused by the reinforced structure would not have too much impact on the system.

**Figure 10:**
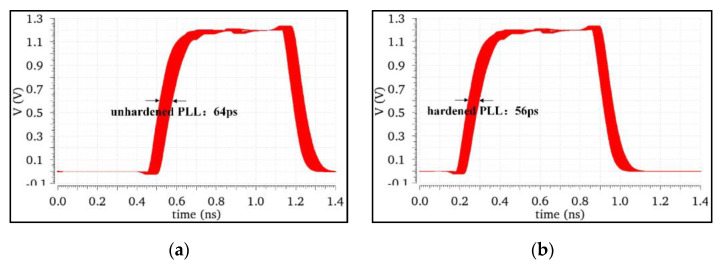
(**a**) Period jitter of the NHPLL. (**b**) Period jitter of the RHPLL.

The size of the clock jitter was determined by the eye diagram of the phase-locked loop output signal. The simulation results of the clock jitter before and after the phase-locked loop hardening are shown in Figure 10. The results showed that the jitter performance of the hardened PLL was better than that of the unhardened PLL. The main reason is that the transmission gate TG1 in the hardened structure had good noise isolation effect, resulting in the better noise performance of the hardened PLL. As a result, the clock jitter of the hardened PLL output signal was also reduced in the time domain.

### 4.2. Verification of Hardened Effect

In the transistor level circuits of the RHPLL and NHPLL, double-exponential current pulses were used to simulate the SET effect to verify the effectiveness of the hardened structure. Through simulation, the peak value of the control voltage fluctuation $\Delta V_{ctrl}$, recovery time $T_{rec}$, maximum phase shift $\Delta \phi_{max}$, and number of output signal error pulses were obtained to measure the SET suppression effect of the hardened structure.

**Figure 11:**
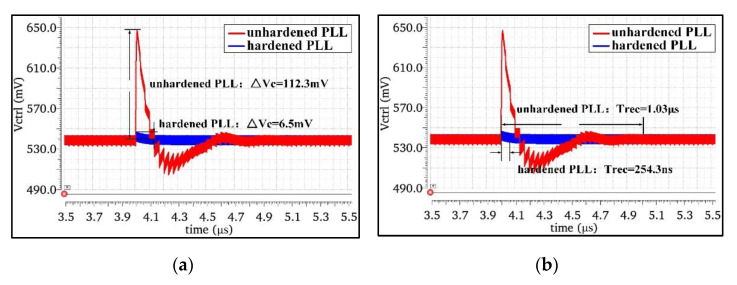
(**a**) Simulation results of the voltage disturbance. (**b**) Simulation results of the recovery time.

When the phase-locked loop was in the fully locked state, the impact of the high-energy particles on the most sensitive node C4 of CP was simulated by adding a SET current source. The charge pump output node C4 of the NHPLL and RHPLL was set to be bombarded by high-energy particles with deposition charges of 0.5 pC at 800 MHz, and the simulation results of the voltage disturbance and recovery time of the output node C4 of the two CPs were obtained, as shown in Figure 11. The results showed that the voltage disturbance $\Delta V_{ctrl}$ of the NHPLL affected by the SET was 112.3 mV, while that of the RHPLL was only 6.5 mV. Compared with the NHPLL, the RHPLL increased the inhibition of SET by 94.2%. The recovery time $T_{rec}$ of the NHPLL to restore the locked state under the influence of the SET was 1.03 μs, while the recovery time $T_{rec}$ of the RHPLL was only 254.3 ns. Compared with the NHPLL, the time required for RHPLL to restore the locked state was reduced by 75.3%.

**Figure 12:**
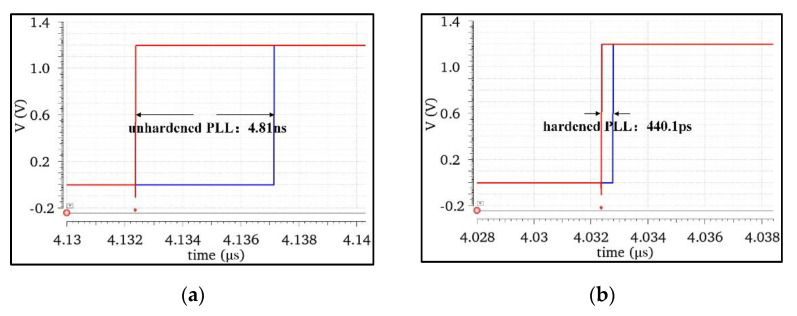
Simulation results of the maximum phase displacement before and after hardening: (**a**) maximum phase shift before hardening; (**b**) maximum phase shift after hardening.

When the phase-locked loop was in the fully locked state, the charge pump output node C4 of the NHPLL and RHPLL was set to be bombarded by high-energy particles with deposition charges of 0.5 pC at 800 MHz, and the simulation results of the comparison between the input reference signal Fref and the feedback signal Ffb of the two were obtained as shown in Figure 12. Formula 2 is the calculation formula of the maximum phase shift, where Tclk = 20 MHz is the period of the input reference signal. The simulation results and calculation showed that the maximum time shift of the NHPLL under the influence of the SET was 4.81 ns, and the maximum phase shift $\Delta \phi_{max}$ was 86.58°. The maximum time shift of the RHPLL was 0.44 ns, and the maximum phase shift $\Delta \phi_{max}$ was 7.93°. Compared with the NHPLL, the maximum phase shift caused by the SET in the RHPLL decreased by 90.8%. We determined the error pulse number of the PLL output signal from the bombardment start time to the normal state time. There were seven error pulses in the NHPLL and two error pulses in the RHPLL. Compared with the NHPLL, the number of error pulses caused by the SET in the RHPLL decreased by 71.43%.

(2) $$\Delta \phi_{\max}=360{}^{\circ}\times \frac{t_{error}}{T_{clk}}$$

Compared with the NHPLL, the RHPLL used the single-event-hardened charge pump structure proposed in this study to achieve a lower voltage disturbance $\Delta V_{ctrl}$, a faster recovery time $T_{rec}$, a fewer number of error pulses, and a smaller maximum phase shift $\Delta \emptyset_{max}$, which effectively reduced the sensitivity of CP output node to the single-event effect and improved the radiation resistance of the PLL.

In order to further verify whether the charge pump reinforcement scheme deteriorated the performance of the PLL, this paper conducted an overall performance simulation before and after the hardening of the PLL and obtained the performance index data, as shown in Table 1. The simulation data showed that the frequency output range, charge pump current, charge–discharge current mismatch rate, control voltage ripple, locking time of the NHPLL and RHPLL, power consumption, and the area were almost the same. The reason for this is that the proposed hardened circuit only worked under the SET bombardment. When the PLL worked normally, the hardened circuit did not affect the performance of the PLL. At the same time, the irradiation resistance of the RHPLL was greatly improved, effectively improving the reliability of the PLL in the irradiation environment.

Table 2 lists the comprehensive comparison between the hardened charge pump structure proposed in this study and the advanced charge pump against the SET effect in other studies. Compared with the other literature, the largest advantage of the hardened structure proposed in this study is that it did not change the loop parameters, reducing the design difficulty, and at the same time, it was better than the other structures in controlling the peak value of the voltage fluctuation.

## 5. Conclusions

In order to improve the reliability of the phase-locked loop microsystem applied in the aerospace environment, a hardened scheme of a phase-locked loop to suppress the single-event effect was proposed in this work. A digital control circuit was added between the traditional charge pump and the low-pass filter, which greatly reduced the sensitivity of the charge pump to the single-event transient and improved the ability of the phase-locked loop system to suppress the single-event effect. The simulation results based on the SMIC 130 nm standard CMOS showed that the fluctuation peak value of the phase-locked loop transient response using the single-event-hardened scheme proposed in this work was reduced by 94.2%, the lock recovery time was improved by 75.3%, and the maximum phase shift was reduced by 90.8%. This shows that the optimization scheme can effectively reduce the sensitivity of the PLL to the SET effect and improve the reliability of the PLL in the irradiation environment.

## Figures and Tables

**Table 1:** Performance comparison of the PLL before and after hardening.

Performance Parameter		NHPLL	RHPLL
Supply voltage		1.2 V	1.2 V
Output frequency range		0.6 GHz~1.6 GHz	0.6 GHz~1.6 GHz
CP working current		25 μA	25 μA
CP current mismatch		0.65%	0.67%
Control voltage ripple		8 mV	8 mV
Lock time@0.8 GHz		2.3 μs	2.5 μs
Power consumption		8.56 mW	9.12 mW
Area		265 μm × 199 μm (1×)	275 μm × 207 μm (1.08×)
SET response	Vctrl fluctuation peak	112.3 mV	6.5 mV
	Recovery time	1031 ns	254.3 ns
	Number of error pulses	7	2
	Maximum phase error	86.58°	7.93°

**Table 2:** Comparison with other work.

Parameter	Reference [14]			Reference [21]			This Work		
Technology node	130 nm CMOS			130 nm CMOS			130 nm CMOS		
Frequency	700 MHz			850 MHz			800 MHz		
Deposited charge or LET	200 fC(≈20 MeVcm^2^/mg)			500 fC(≈48 MeVcm^2^/mg)			500 fC(≈48 MeVcm^2^/mg)		
Loop parameters	Redesign			Redesign			No change		
Hardened or not	unhardened	hardened	improvement	unhardened	hardened	improvement	unhardened	hardened	improvement
Voltage perturbation $\Delta V_{ctrl}\left(mV\right)$	640	42	93%	85	13	84.7%	112.3	6.5	94.2%
Recovery time $T_{rec}\left(ns\right)$	400	98	75.5%	340	114	66.5%	1031	254.3	75.3%

## Data Availability

Not applicable.

## References

[1] von Kaenel V.R. (1998). A high-speed, low-power clock generator for a microprocessor application. IEEE J. Solid-State Circuits.

[2] Chen Z., Ding D., Dong Y., Shan Y., Zeng Y., Gao J. (2020). Design of a High-Performance Low-Cost Radiation-Hardened Phase-Locked Loop for Space Application. IEEE Trans. Aerosp. Electron. Syst..

[3] Grubin H.L., Kreskovsky J.P., Weinberg B.C. (1984). Numerical Studies of Charge Collection and Funneling in Silicon Device. IEEE Trans. Nucl. Sci..

[4] Van Dam C., Hauser M. Ring oscillator reliability model to hardware correlation in 45nm SOI. Proceedings of the 2013 IEEE International Reliability Physics Symposium (IRPS).

[5] Alaoui Z., Alaoui I., Ajib E.W., Nabki F., Gagnon F. (2021). A 0.1–9-GHz Frequency Synthesizer for Avionic SDR Applications in 0.13-μm CMOS Technology. IEEE Trans. Very Large Scale Integr. (VLSI) Syst..

[6] Rossetto A.C., Wirth G.I., Dallasen R.V. Performance analysis of a clock generator PLL under TID effects. Proceedings of the 2014 15th Latin American Test Workshop—LATW.

[7] Boulghassoul Y., Massengill L.W., Sternberg A.L., Bhuva B.L., Holman W.T. (2006). Towards SET Mitigation in RF Digital PLLs: From Error Characterization to Radiation Hardening Considerations. IEEE Trans. Nucl. Sci..

[8] Chen Z., Lin M., Zheng Y., Wei Z., Huang S., Zou S. (2016). Single-Event Transient Characterization of a Radiation-Tolerant Charge-Pump Phase-Locked Loop Fabricated in 130 nm PD-SOI Technology. IEEE Trans. Nucl. Sci..

[9] Chen Z., Lin M., Zheng Y., Wei Z., Huang S., Zou S. (2017). Analysis of Single-Event Effects in a Radiation-Hardened Low-Jitter PLL Under Heavy Ion and Pulsed Laser Irradiation. IEEE Trans. Nucl. Sci..

[10] Loveless T.D., Massengill L.W., Bhuva B.L., Holman W.T. (2007). A Single-Event-Hardened Phase-Locked Loop Fabricated in 130 nm CMOS. IEEE Trans. Nucl. Sci..

[11] Loveless T.D., Olson B.D., Bhuva B.L., Holman W.T., Hafer C.C. (2009). Analysis of Single-Event Transients in Integer-N Frequency Dividers and Hardness Assurance Implications for Phase-Locked Loops. IEEE Trans. Nucl. Sci..

[12] Richards E.W., Loveless T.D., Kauppila J.S., Haeffner T.D., Holman W.T., Massengill L.W. (2020). Radiation Hardened by Design Subsampling Phase-Locked Loop Techniques in PD-SOI. IEEE Trans. Nucl. Sci..

[13] Liu H., Zhang X., Li P., Xu X. A Hardened Phase-Locked Loop Using Novel Charge Pump. Proceedings of the 2015 8th International Symposium on Computational Intelligence and Design (ISCID).

[14] Liang B., Xu X., Yuan H., Chen J. (2022). A system-level method for hardening phase-locked loop to single-event effects. Mater. Res. Express.

[15] Loveless T.D., Massengill L.W., Bhuva B.L. (2006). A Hardened-by-Design Technique for RF Digital Phase Lock Loops. IEEE Trans. Nucl. Sci..

[16] Zhao Z., Zhang M., Chen S., Chen J., Li J. (2009). A radiation-hardened-by-design technique for improving single-event transient tolerance of charge pumps in PLLs. J. Semicond..

[17] Han B., Guo Z., Wu L., Liu Y. (2012). A single-event-hardened phase-locked loop using the radiation-hardened-by-design technique. J. Semicond..

[18] Adesina N.O., Srivastava A., Khan M.A.U., Xu J. Phase Noise and Jitter Measurements in SEU-Hardened CMOS Phase Locked Loop Design. Proceedings of the 2021 IEEE International IOT, Electronics and Mechatronics Conference (IEMTRONICS).

[19] Messenger G.C. (1982). Collection of charge on junction nodes from ion tracks. IEEE Trans. Nucl. Sci..

[20] Casey M.C., Bhuva B.L., Black J.D. (2005). HBD using cascode-voltage switch logic gates for SET tolerant digital designs. IEEE Trans. Nucl. Sci..

[21] You Y., Chen J., Feng Y. (2015). SET detection and compensation and its application in PLL design. J. Instrum..

